# Peritumoral edema shown by MRI predicts poor clinical outcome in glioblastoma

**DOI:** 10.1186/s12957-015-0496-7

**Published:** 2015-03-11

**Authors:** Chen-Xing Wu, Guo-Shi Lin, Zhi-Xiong Lin, Jian-Dong Zhang, Shui-Yuan Liu, Chang-Fu Zhou

**Affiliations:** Department of Neurosurgery, Beijing Sanbo Brain Hospital, Capital Medical University, Beijing, 100093 China; Department of Neurosurgery, The First Affiliated Hospital of Fujian Medical University, 20 Cazhong Road, Fuzhou, Fujian 350005 China

**Keywords:** Glioblastoma, Prognosis, Peritumoral edema, Necrosis, MRI

## Abstract

**Background:**

Magnetic resonance imaging (MRI) plays an irreplaceable role in the preoperative diagnosis of glioma, and its imaging features are the base of making treatment decisions in patients with glioma, but it is still controversial whether peritumoral edema shown by MRI from preoperative routine scans are associated with patient survival. The aim of this study was to assess the prognostic value of preoperative MRI features in patients with glioblastoma.

**Methods:**

A retrospective review of 87 patients with newly diagnosed supratentorial glioblastoma was performed using medical records and MRI data from routine scans. The Kaplan-Meier method and COX proportional hazard model were applied to evaluate the prognostic impact on overall survival of pretreatment MRI features (including peritumoral edema, edema shape, necrosis, cyst, enhancement, tumor crosses midline, edema crosses midline, and tumor size).

**Results:**

In addition to patient age, Karnofsky performance status (KPS) and postoperative chemoradiotherapy, peritumoral edema extent and necrosis on preoperative MRI were independent prognostic indicator for poor survival. Furthermore, patients with two unfavorable conditions (major edema and necrosis) had a shorter overall survival compared with the remainder.

**Conclusions:**

Our data confirm that peritumoral edema extent and necrosis are helpful for predicting poor clinical outcome in glioblastoma. These features were easy to determine from routine MRI scans postoperatively and therefore could provide a certain instructive significance for clinical activities.

## Background

Glioblastoma multiforme (GBM) is the most prevalent malignant brain tumor in adults and accounts for 17% of intracranial tumors [1]. To date, effective durable treatments in patients with GBM are still lacking, exhibiting a poor prognosis with a median overall survival rate of 9.4 to 19.0 months despite advances in multimodal treatments that combine surgery, radiation therapy, and chemotherapy [2].

Magnetic resonance imaging (MRI) technology is a common and noninvasive diagnostic modality and was previously found to take examination in central nervous system disease in clinical and especially in the preoperative diagnosis of glioma. Moreover, MRI is not only a powerful tool to visualize changes in morphological abnormalities but also a direct reflection of biochemical changes in the tumor and surrounding tissue. MRI can have great utility in the diagnosis, grading, and management of patients with GBM as many of the physical manifestations of the pathologic processes in GBM can be visualized and quantified using MRI. Better taking account of the correlation between preoperative tumor imaging features and survival is therefore useful to clinic.

For patients with GBM, clinical data-including age, perioperative Karnofsky performance status (KPS), pathological molecular markers, tumor resection, adjuvant radiochemotherapy, and tumor imaging features (including necrosis and edema) have been found to correlate with survival [3-7]. Moreover, a clear relationship among survival pattern with peritumoral edema and necrosis in GBM has not been established. Some tumor imaging features of preoperative MRI from conventional scans, such as peritumoral edema (PTE) extent, necrosis, enhancement, and the size of cyst, were considered to be correlated with patient survival. [5,8-11]. However, several reports showed that these features, such as PTE and necrosis, were not independent values of survival in patients with glioblastoma [12-17].

These controversial results therefore suggest that there remains a need to further evaluate whether PTE and necrosis on MRI are associated with patient survival because such data from routine imaging play an irreplaceable role during preoperative diagnosis and now are the kernel base of making treatment decisions in patients with glioma, clearly recognizing the relationship among them has a certain instructive significance for clinical practice. Here, the tumor imaging features (including PTE, edema shape, necrosis, cyst, enhancement, tumor crosses midline, edema crosses midline, and tumor size) from preoperative routine MRI scans were assessed, and the aim of our study was to examine whether these characters are more valuable prognostic markers in patients with primary glioblastoma.

## Methods

### Study samples

Clinical and preoperative MRI data of 87 patients treated with resection of newly diagnosed supratentorial GBM at Beijing Sanbo Brain Hospital of Capital Medical University between April 2009 and March 2013 were introduced into this retrospective study. The exclusion criteria in this study were as follows: i) patients who died of non-glioma cause, ii) patients who received biopsy were excluded from the study, ii) patients who received Corticosteroids at the time of the preoperative MRI scan. For all patients enrolled in the study, the tumor was confirmed to be totally resected using postoperative enhanced MRI within 3 days. According to the principles of WHO classification [1], the histological diagnosis of each patient was reaffirmed. Postoperatively, radiotherapy (plus concurrent temozolomide chemotherapy at a dose of 75 mg/M^2^/day) was administered in contrast-enhanced lesion plus the area of PTE and a 2-cm margin (60 Gy in 2 Gy fractions). Then, temozolomide chemotherapy (150 to 200 mg/M^2^/day) was administered for six cycles unless death or irreversible hematological toxicity occurred. All patients were followed up through either telephone or outpatient visit. Overall survival (OS) was defined as the time interval (days) between primary surgical resection and death (or the latest follow-up). This study was approved by the local Ethics Committee and was conformed to the principles outlined in Declaration of Helsinki. Written informed consent was provided by all patients.

### Classification of MRI features

**Figure 1:**
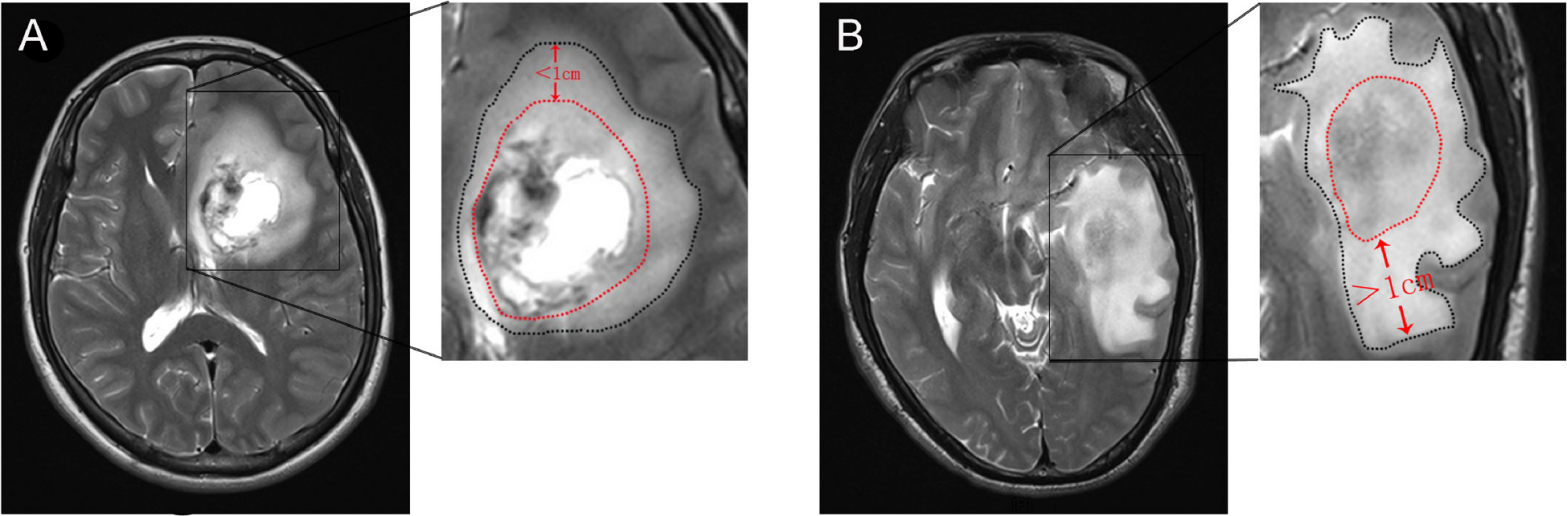
**Eveluation of PTE.** A region of very bright T2-W signal surrounding the tumor, which was estimated on the base of the maximum distance from the tumor margin to the outer edge of edema. **(A)** Minor edema (<1 cm) shown by T2-W MRI. **(B)** Major edema (>1 cm) shown by T2-W MRI.

For all patients, preoperative MRI data from routine scans (1.5-T scanner) including T1-W, T2-W, and contrast-enhanced T1-W sequences were available. The unidimensional maximum diameter in centimeters was used for measuring tumor size on T1-W images; median tumor size was 5.0 cm (rang 2.3 to 9.9 cm). The region of very bright T2-W signal surrounding the tumor was defined as PTE, which was estimated on the base of the maximum distance from the tumor margin to the outer edge of edema and was graded as follows [6]: minor (Figure 1A) and major (Figure 1B). According to the method of Hartmann [18], the morphological classification of PTE was performed on the base of T2-W images. Necrosis which was estimated on axial contrast-enhanced T1-W images [19] was determined when a region had high signal on T2-W images, but low signal on T1-W images, and had an irregular enhancing border on contrast-enhanced images. Cyst was defined as a rounded region which was low T1-W signal and very high T2-W signal matching cerebrospinal fluid (CSF) signal and had a thin, smooth, regular, and slightly enhancing or non-enhancing wall [10]. Contrast enhancement in tumor was grouped as no obvious (enhancement signal is less than the signal of fat) and obvious (enhancement signal is similar to that of fat). The specific classification of imaging features was listed in Table 1. According to the classification methods mentioned above, imaging data of all patients were analyzed independently by two experienced radiologists without knowledge of patient clinical information.

**Table 1:** **Specific classification of MRI features**

**Imaging features**	**Classification criterion**
Edema extent	
Minor	Peritumoral edema extending <1 cm from tumor margin
Major	Peritumoral edema extending ≥1 cm from tumor margin
Edema shape	
Roundish	The shape of edema is similar to round and is not radial
Irregular	The shape of edema tends to irregular, such as finger-like or radial shape
Necrosis	
No	No necrosis within tumor
Yes	A region had high signal on T2-W images, but low signal on T1-W images, and had an irregular enhancing border on contrast-enhanced images
Cyst	
No	No cyst in tumor
Yes	A rounded region which was very low T1-W signal and very high T2-W signal matching cerebrospinal fluid signal, and had a thin, smooth, regular, and slightly enhancing or non-enhancing wall
Enhancement	
No obvious	Enhancement signal is less than the signal of fat
Obvious	Enhancement signal is similar to that of fat
Tumor crosses midline	
No	Tumor is limited to unilateral cerebral hemisphere
Yes	Tumor crosses the brain midline and extends into the other side of cerebral hemisphere
Edema crosses midline	
No	Peritumoral edema extent is limited to unilateral cerebral hemisphere
Yes	Peritumoral edema extent crosses the brain midline and is not confined to unilateral cerebral hemisphere
Size (cm)	
<5	The maximum diameter of tumor is less than 5 cm
≥5	The maximum diameter of tumor is equal to or more than 5 cm

### Statistical analysis

SPSS 19.0 was applied to statistical analysis. In univariate analysis, the Kaplan-Meier method was used to calculate survival rates which were compared by the log-rank test. COX proportional hazard model and stepwise regression analysis were applied to estimate the influence of preoperative MRI features on survival in multivariate analysis. Hazard ratios (HRs) and their 95% confidence intervals (CIs) were also computed. *P =* 0.05 (two-sided) was considered statistically significant.

## Results

### Clinical characteristics

Out of 87 cases, 55 (63.2%) patients were male and 32 (63.2%) were female. Median age at diagnosis was 60 years (range 25 to 78). Median preoperative KPS of patients was 80 (range 70 to 100). Postoperatively, forty-three patients were treated with radiotherapy plus chemotherapy, which was determined as standard chemoradiotherapy; twenty-nine patients had radiotherapy; eleven patients had chemotherapy alone; and four patients refused radiotherapy and chemotherapy. For statistical analysis, these patients were defined as nonstandard chemoradiotherapy.

The main distribution of patient characteristics in PTE subgroups is summarized in Table 2. A statistically significant correlation emerged among edema extent with edema shape (*R* = 0.570, *P* = 0.000) and enhancement (*R* = 0.436, *P* = 0.000). No significant correlation was found between edema grade and gender, age, KPS, necrosis, cyst, tumor crosses midline, edema crosses midline, and tumor size.

**Table 2:** **Main distribution of patient characteristics in peritumoral edema subgroup**

**Variables**	**Number of cases (%)**	**Peritumoral edema extent**	
		**Minor edema (%)**	**Major edema (%)**
Total	87	19 (21.8)	68 (78.2)
Gender			
Male	55 (63.2)	14 (73.7)	41 (60.3)
Female	32 (36.8)	5 (26.3)	27 (39.7)
Age (years)			
≥60	50 (57.5)	12 (63.2)	38 (55.9)
<60	37 (42.5)	7 (36.8)	30 (44.1)
KPS			
≤80	33 (37.9)	5 (26.3)	28 (41.2)
>80	54 (62.1)	14 (73.7)	40 (58.8)
Edema shape			
Roundish	29 (33.3)	16 (84.2)	13 (19.1)
Irregular	58 (66.7)	3 (15.8)	55 (80.9)
Necrosis			
No	16 (18.4)	6 (31.6)	10 (14.7)
Yes	71 (81.6)	13 (68.4)	58 (85.3)
Cyst			
No	67 (77)	15 (78.9)	52 (75.4)
Yes	20 (23)	4 (21.1)	16 (24.6)
Enhancement			
No obvious	30 (34.5)	14 (73.7)	16 (23.5)
Obvious	57 (65.5)	5 (26.3)	52 (76.5)
Tumor crosses midline			
No	71 (81.6)	17 (89.5)	54 (79.4)
Yes	16 (18.4)	2 (10.5)	14 (20.6)
Edema crosses midline			
No	59 (67.8)	17 (89.5)	42 (61.8)
Yes	28 (32.2)	2 (10.5)	26 (38.2)
Size (cm)			
<5	37 (42.5)	6 (31.6)	31 (45.6)
≥5	50 (57.5)	13 (68.4)	37 (54.4)

### Survival analysis

**Figure 2:**
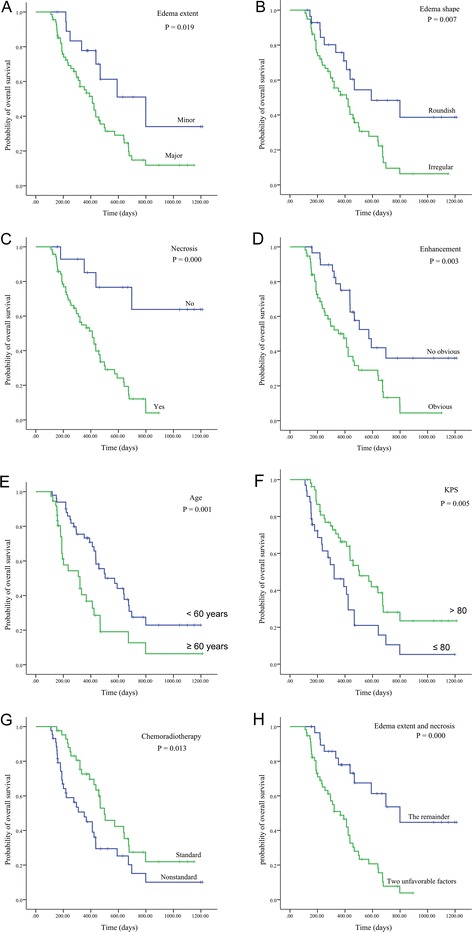
**Kaplan-Meier curves.** Kaplan-Meier curves showing correlations of PTE **(A)**, edema shape **(B)**, enhancement necrosis **(C)**, enhancement **(D)**, age **(E)**, KPS **(F)**, chemoradiotherapy **(G)**, and major PTE and necrosis **(H)** with overall survival in the entire cohort.

To evaluate the influence of on prognosis, telephone or outpatient visit was applied in the entire cohort (followed up time minimum 101 days, maximum 1,198 days, median 352 days), and corresponding OS was calculated. Out of 87 patients, the median OS was 435 days (95% CI 374 to 495) in the entire cohort. Univariate analysis (Table 3) revealed major PTE was shown to be significantly associated with a dismal OS (*P* = 0.019, Figure 2A) and patients with minor PTE exhibited longer survival compared with major edema. Additionally, similar results were obtained for edema shape (*P* = 0.007, Figure 2B), necrosis (*P* = 0.000, Figure 2C), enhancement (*P* = 0.003, Figure 2D), patient age (*P* = 0.001, Figure 2E), KPS (*P* = 0.005, Figure 2F), and chemoradiotherapy (*P* = 0.013, Figure 2G). However, no significant difference was observed among OS with gender, cyst, tumor crosses midline, edema crosses midline, and tumor size (*P* > 0.05).

**Table 3:** **Variables associated with the overall survival in the entire cohort: univariate analysis**

**Variables**	**Number of cases**	**Overall survival (days)**		***P*** **-value**
		**Median**	**95% CI**	
Total	87	435	374 to 495	
Gender				
Male	55	437	372 to 501	0.723
Female	32	423	362 to 484	
Age (years)				
≥60	50	321	166 to 473	0.001
<60	37	504	327 to 681	
KPS				
≤80	33	321	187 to 455	0.005
>80	54	504	323 to 684	
Chemoradiotherapy				
Standard	43	498	369 to 627	0.013
Nonstandard	44	352	229 to 474	
Edema extent				
Minor	19	599	397 to 956	0.019
Major	68	411	332 to 489	
Edema shape				
Roundish	29	591	270 to 962	0.007
Irregular	58	411	283 to 539	
Necrosis				
No	16	687	476 to 856	0.000
Yes	71	410	311 to 510	
Cyst				
No	67	436	382 to 490	0.73
Yes	20	391	206 to 575	
Enhancement				
No obvious	30	573	412 to 734	0.003
Obvious	57	391	275 to 507	
Tumor crosses midline				
No	71	436	382 to 490	0.73
Yes	16	391	206 to 575	
Edema crosses midline				
No	59	458	417 to 498	0.153
Yes	28	321	212 to 430	
Size (cm)				
<5	37	391	226 to 527	0.305
≥5	50	458	263 to 652

Factors which were statistically significant from univariate analysis were introduced into multivariate analysis. Multivariate survival analysis (Table 4) demonstrated major edema and necrosis on MRI as significant prognostic indicators for shorter OS (HR 2.274, *P* = 0.015; HR 2.215, *P* = 0.001, respectively). Likewise, advanced age at diagnosis (≥60 years), poor performance status (≤80), and nonstandard chemoradiotherapy were also confirmed to be independent predictors for poor OS.

**Table 4:** **Statistically significant prognosis indicators evaluated by multivariate analysis in the entire cohort**

**Variables**	**Hazard ratio (HR)**	**95% CI**	***P*** **-value**
Edema extent	2.274	1.238 to 5.991	0.015
Necrosis	2.215	1.447 to 3.401	0.001
Age	1.954	1.137 to 3.358	0.029
KPS	0.502	0.292 to 0.864	0.013
Chemoradiotherapy	0.358	0.204 to 0.630	0.000

Based on the above analysis, we investigated whether patients with two unfavorable factors (major edema and necrosis) had a shorter survival compared with those with only one or without unfavorable factor (either major edema or necrosis). The statistical results revealed that the prognosis in patients with two unfavorable factors was obviously poorer than the remainder (*P* = 0.000, Figure 2H). When adjusted for patient age, KPS, and chemoradiotherapy and the adjusted HR was 1.0 (as a reference), the adjusted HR of patients with two unfavorable factors was 5.031 (95% CI 2.449 to 10.338, *P* = 0.000) for OS.

## Discussion

While the prognosis for patients with GBM is relatively poor, variability of survival among patients who are given the same malignant level suggests that there are additional underlying factors that influence how the tumor progresses. The main causes of morbidity and mortality in glioma patients is the induction of severe cerebral edema and necrosis, which lead to brain herniation in up to 60% of patients with GBM [20], but some study suggested that there is still a controversy about these prognostic values [15,16,18]. Therefore, the relationship between survival and the appearance of tumor on MRI is important. In our retrospective study, we found that PTE and necrosis were statistically significant unfavorable prognosis factors affecting OS in patients with newly diagnosed supratentorial GBM.

PTE, the abnormal accumulation of water inside the brain parenchyma, is commonly seen in GBM patients [16]. Our study found that edema was significant prognostically in both univariate and multivariable analysis, as others have reported. Patients with minor PTE exhibited longer survival compared with major edema. Multivariate survival analysis demonstrated major edema on MRI as independent prognostic indicators for shorter OS (HR 2.274, *P* = 0.015, respectively). Based on the literature, we hypothesized that it may be related to the fact that the tumor cells infiltrate the peritumoral-edema areas [21]. Second off, glioblastoma is associated with infiltration of peritumoral parenchyma by isolated tumor cells that leads to tumor regrowth. Recently, GBM stem-like or initiating cells (GICs) have been identified in the peritumoral areas [22], and these GICs have enhanced migratory and invasive capabilities compared with GICs from the tumor mass, which are the sources of tumor recurrence [23-25]. Moreover, peri-tumoral edematous fluid can accumulate rapidly in severe cases [26]. Within the rigid skull, rapid augmentation of brain volume leads to a sharp increase in ICP, which can result in decreased cerebral blood flow, ischemia, brain herniation, and death [26]. In this study, the extent of edema turned out to be an independent prognostic factor in patients with GBM, which confirms the results of Pope WB and K Schoenegger. However, in the literature, results concerning the prognostic impact of brain edema in glioblastoma patients have not been conclusive and uniform. Other studies either found that there was no relationship between these variables [10,12,14] or that the relationship varied depending on the extent of the edema [11]. Many remarkable factors contribute to the explanations, firstly, given the nature of the studies included was retrospective and the between-study heterogeneity in terms of patient clinical characteristics and the imaging technology used about the topic, another factor which was inconsistent among these studies in evaluation and definition of PTE [15]. Our study also showed that a statistically significant correlation emerged among edema extent with edema shape (*R* = 0.570, *P* = 0.000) and enhancement (*R* = 0.436, *P* = 0.000); the patients with irregular shape of edema and obvious enhancement survived shorter than those with vice versa, but multivariate analysis indicated that edema shape and enhancement were both not an independent predictor of prognosis. It is thought that blood–brain barrier break downs and typically lacks endothelial tight junctions in GBM, which leads to both enhancement and edema. In addition, the perifocal edema is proposed as the clinical target volume, since it has been supposed to contain infiltrating tumor cells, which spread to white matter fiber, causing the irregular shape of edema (such as radial or finger-like shape), both ultimately influences patient survival.

Necrosis is one of the radiologic and pathological characteristics of glioblastoma; in particular, the absence of necrosis on imaging studies was an important prognostically favorable variety, confirming the findings of Hammoud *et al*. Necrotic areas within a glioma are a common imaging feature and are believed to indicate rapid growth and malignant behavior. Previous studies have conflicted that tumor necrosis was related to survival in GBM [9,26,27]. In a series of 75 glioblastoma patients, David A. Gutman *et al*. found contrast-enhanced tumor volume and longest axis length of tumor were strongly associated with poor survival. While necrosis was not found to be an independent prognostic factor [28].In the present study, necrosis on preoperative MRI was found to be an independent prognostic factor in multivariate analysis (HR 2.215, *P* = 0.001, respectively). One proposed explanation is that the rapid cellular proliferation of tumor cells brings about nutrient unbalance, which leads to hypoxia and until necrosis in tumor tissue. Hypoxia selects for cells with diminished apoptotic potential relative to those in the original cell population. Moreover, necrosis and hypoxia in gliomas lead to upregulation of vascular endothelial growth factor expression that stimulates angiogenesis [29,30], thereby making the tumor cell population peripheral to the necrosis, improving the invasion ability and resistance to radiochemotherapy [31-35].

Previous studies showed that enhancement on preoperative contrast T1-weight MRI was an independent value of survival in GBM [9,25]. However, in this study, enhancement was related to OS of GBM in univariate analysis while not same in multivariate analysis. According to the hypothesized pathways that are present in the study, the enhancement of tumors mainly reflects the destruction of blood–brain barrier maintenance and is influenced by all processes that decrease or increase the abnormal permeability, in spite of the size and activity of tumor [36]. It was previously thought that cysts were associated with improved outcome [37-39]. It is probably that the etiology of cyst formation implies more indolent tumor growth [13]. Nevertheless, our study demonstrates that the presence of cystic features does not confer a survival advantage, which is in accordance with a recent large-series study [13]. However, the exact mechanism of cyst formation remains unclear and needed to be further researched.

In addition to the clinical characteristics of age and preoperative KPS score, for which the relationship to survival is well established [4,5], we observed the same results in our series, advanced age at diagnosis (≥60 years), poor performance status (≤80), and nonstandard chemoradiotherapy were also confirmed to be independent predictors for poor OS (HR 1.954, *P* = 0.029; HR 0.502, *P* = 0.029, respectively). We deduce that it might be due to the biological characteristics and behaviors of glioma cells of patient at different age group. Moreover in our study, postoperative standard radio-chemotherapy could prolong the survival time for patients with GBM (HR 0.358, *P* = 0.000, respectively), which is in accordance with previous studies [3,40]; thus, it is advocated actively to take standard therapy regimen for patients with GBM postoperatively.

However, it should be noted that in this study, many inherently limitations exist. Obviously, this is a retrospective-design research which might be inevitably subject to bias that not all can be controlled for in this context. Furthermore, limitations of the study include small sample size. In the future, to further disclose the key molecular mechanisms of those independent predictors of survival, large-scale and prospective studies are needed.

## Conclusions

In summary, PTE extent and necrosis shown by MRI from preoperative routine scans are independent unfavorable prognosis indicators, and a patient with both major edema and necrosis exhibits a poorer prognosis, thereby indicating that PTE extent and necrosis which are easy to be determined from routine MRI scans can be used to predict OS in patients with newly diagnosed GBM.

## Abbreviations

GBM: Glioblastoma multiform
PTE: Peritumoral edema
MRI: Magnetic resonance imaging
OS: Overall survival
KPS: Karnofsky performance status
CSF: Cerebrospinal fluid
HR: Hazard ratio
GICs: Stem-like or initiating cells

## References

[1] Louis DN, Ohgaki H, Wiestler OD, Cavenee WK, Burger PC, Jouvet A (2007). The 2007 WHO classification of tumours of the central nervous system. Acta Neuropathol.

[2] Central Brain Tumor Registry of the United States (2010). CBTRUS statistical report: primary brain and central nervous system tumors in the United States in 2004–2006.

[3] Yang LJ, Zhou CF, Lin ZX (2014). Temozolomide and radiotherapy for newly diagnosed glioblastoma multiforme: a systematic review. Cancer Invest..

[4] Buckner JC (2003). Factors influencing survival in high-grade gliomas. Semin Oncol..

[5] Dahlrot RH (2014). The prognostic value of clinical factors and cancer stem cell-related markers in gliomas. Dan Med J.

[6] Schoenegger K, Oberndorfer S, Wuschitz B, Struhal W, Hainfellner J, Prayer D (2009). Peritumoral edema on MRI at initial diagnosis: an independent prognostic factor for glioblastoma?. Eur J Neurol..

[7] Das P, Puri T, Jha P, Pathak P, Joshi N, Suri V (2011). A clinicopathological and molecular analysis of glioblastoma multiforme with long-term survival. J Clin Neurosci..

[8] Stupp R, Hegi ME, Gorlia T, Erridge SC, Perry J, Hong YK (2014). Cilengitide combined with standard treatment for patients with newly diagnosed glioblastoma with methylated MGMT promoter (CENTRIC EORTC 26071–22072 study): a multicentre, randomised, open-label, phase 3 trial. Lancet Oncol..

[9] Hammoud MA, Sawaya R, Shi W, Thall PF, Leeds NE (1996). Prognostic significance of preoperative MRI scans in glioblastoma multiforme. J Neurooncol..

[10] Pope WB, Sayre J, Perlina A, Villablanca JP, Mischel PS, Cloughesy TF (2005). MR imaging correlates of survival in patients with high-grade gliomas. AJNR Am J Neuroradiol..

[11] Maldaun MV, Suki D, Lang FF, Prabhu S, Shi W, Fuller GN (2004). Cystic glioblastoma multiforme: survival outcomes in 22 cases. J Neurosurg..

[12] Carrillo JA, Lai A, Nghiemphu PL, Kim HJ, Phillips HS, Kharbanda S (2012). Relationship between tumor enhancement, edema, IDH1 mutational status, MGMT promoter methylation, and survival in glioblastoma. Am J Neuroradiol..

[13] Kaur G, Bloch O, Jian BJ, Kaur R, Sughrue ME, Aghi MK (2011). A critical evaluation of cystic features in primary glioblastoma as a prognostic factor for survival. J Neurosurg..

[14] Pierallini A, Bonamini M, Osti MF, Pantano P, Palmeggiani F, Santoro A (1996). Supratentorial glioblastoma: neuroradiological findings and survival after surgery and radiotherapy. Neuroradiology..

[15] Liu SY, Mei WZ, Lin ZX (2013). Pre-operative peritumoral edema and survival rate in glioblastoma multiforme. Onkologie..

[16] Lin ZX (2013). Glioma-related edema: new insight into molecular mechanisms and their clinical implications. Chin J Cancer..

[17] Bonavia R, Inda MM, Cavenee WK, Furnari FB (2011). Heterogeneity maintenance in glioblastoma: a social network. Cancer Res..

[18] Hartmann M, Jansen O, Egelhof T, Forsting M, Albert FK, Sartor K (1998). Effect of brain edema on the recurrence pattern of malignant gliomas. Radiologe..

[19] Seidel C, Dorner N, Osswald M, Wick A, Platten M, Bendszus M (2011). Does age matter? A MRI study on peritumoral edema in newly diagnosed primary glioblastoma. BMC Cancer..

[20] Silbergeld DL, Rostomily RC, Alvord EC (1991). The cause of death in patients with glioblastoma is multifactorial: clinical factors and autopsy findings in 117 cases of supratentorial glioblastoma in adults. J Neurooncol..

[21] Yamahara T, Numa Y, Oishi T, Kawaguchi T, Seno T, Asai A (2010). Morphological and flow cytometric analysis of cell infiltration in glioblastoma: a comparison of autopsy brain and neuroimaging. Brain Tumor Pathol..

[22] Ruiz-Ontanon P, Orgaz JL, Aldaz B, Elosegui-Artola A, Martino J, Berciano MT (2013). Cellular plasticity confers migratory and invasive advantages to a population of glioblastoma-initiating cells that infiltrate peritumoral tissue. Stem Cells..

[23] Zhang X, Zhang W, Mao XG, Zhen HN, Cao WD, Hu SJ (2013). Targeting role of glioma stem cells for glioblastoma multiforme. Curr Med Chem..

[24] Chen J, Li Y, Yu TS, McKay RM, Burns DK, Kernie SG (2012). A restricted cell population propagates glioblastoma growth after chemotherapy. Nature..

[25] Mangiola A, de Bonis P, Maira G, Balducci M, Sica G, Lama G (2008). Invasive tumor cells and prognosis in a selected population of patients with glioblastoma multiforme. Cancer..

[26] Ito U, Reulen HJ, Tomita H, Ikeda J, Saito J, Maehara T (1990). A computed tomography study on formation, propagation and resolution of edema fluid in metastatic brain tumors. Adv Neurol..

[27] Lacroix M, Abi-Said D, Fourney DR, Gokaslan ZL, Shi W, DeMonte F (2001). A multivariate analysis of 416 patients with glioblastoma multiforme: prognosis, extent of resection, and survival. J Neurosurg..

[28] Gutman DA, Cooper LA, Hwang SN, Holder CA, Gao J, Aurora TD (2013). MR imaging predictors of molecular profile and survival: multi-institutional study of the TCGA glioblastoma dataset. Radiology..

[29] D’Angelo MG, Afanasieva T, Aguzzi A (2000). Angiogenesis in transgenic modelsof multistep carcinogenesis. J Neurooncol..

[30] Plate KH, Breier G, Millauer B, Ullrich A, Risau W (1993). Up-regulation of vascular endothelial growth factor and its cognate receptors in a rat gliomamodel of tumor angiogenesis. Cancer Res..

[31] Pierallini A, Bonamini M, Pantano P, Palmeggiani F, Raguso M, Osti MF (1998). Radiological assessment of necrosis in glioblastoma: variability and prognostic value. Neuroradiology..

[32] Rong Y, Durden DL, Van Meir EG, Brat DJ (2006). 'Pseudopalisading' necrosis in glioblastoma: a familiar morphologic feature that links vascular pathology, hypoxia, and angiogenesis. J Neuropathol Exp Neurol..

[33] Brat DJ, Castellano-Sanchez AA, Hunter SB, Pecot M, Cohen C, Hammond EH (2004). Pseudopalisades in glioblastoma are hypoxic, express extracellular matrix proteases, and are formed by an actively migrating cell population. Cancer Res..

[34] Huang XD, Wang ZF, Dai LM, Li ZQ (2012). Microarray analysis of the hypoxia-induced gene expression profile in malignant C6 glioma cells. Asian Pac J Cancer Prev..

[35] Oliver L, Olivier C, Marhuenda FB, Campone M, Vallette FM (2009). Hypoxia and the malignant glioma microenvironment: regulation and implications for therapy. Curr Mol Pharmacol..

[36] Jyothirmayi R, Madhavan J, Nair MK, Rajan B (1997). Conservative surgery and radiotherapy in the treatment of spinal cord astrocytoma. J Neurooncol..

[37] Shibamoto Y, Kitakabu Y, Takahashi M, Yamashita J, Oda Y, Kikuchi H (1993). Supratentorial low-grade astrocytoma. Correlation of computed tomography findings with effect of radiation therapy and prognostic variables. Cancer..

[38] Adn M, Saikali S, Guegan Y, Hamlat A (2006). Pathophysiology of glioma cyst formation. Med Hypotheses..

[39] Utsuki S, Oka H, Suzuki S, Shimizu S, Tanizaki Y, Kondo K (2006). Pathological and clinical features of cystic and noncystic glioblastomas. Brain Tumor Pathol..

[40] Stupp R, Mason WP, van den Bent MJ, Weller M, Fisher B, Taphoorn MJ (2005). Radiotherapy plus concomitant and adjuvant temozolomide for glioblastoma. N Engl J Med..

